# *Streptobacillus moniliformis *septic arthritis: a clinical entity distinct from rat-bite fever?

**DOI:** 10.1186/1471-2334-7-56

**Published:** 2007-06-11

**Authors:** Teresa KF Wang, Samson SY Wong

**Affiliations:** 1 Department of Microbiology, Research Centre of Infection and Immunology, 4/F University Pathology Building, The University of Hong Kong, Queen Mary Hospital, Pokfulam Road, Hong Kong

## Abstract

**Background:**

*Streptobacillus moniliformis *is a zoonotic agent associated with rodent contacts. Although it is more commonly reported to cause rat-bite fever with reactive arthritides, it can also lead to pyogenic infection of the joints.

**Case presentation:**

We present a lady with past history of osteoarthritis developing streptobacillary septic arthritides of the right knee and left wrist, and required antibiotic and arthrotomy for treatment. We also review 11 previously reported cases of streptobacillary septic arthritis to discuss the characteristics, treatment, prognosis of the infection, and illustrates the differences between streptobacillary rat-bite fever and septic arthritis. Among this patient population, most patients had potential contact with rats (91.6%). The knee is the most commonly affected joint (58.3%), and 83.3% patients having polyarticular involvement. As opposed to rat-bite fever, fever and rash was only present in 58.3% and 16.7% of patients respectively. *S. moniliformis *bacteremia is uncommon (8.4%) and the prognosis is good.

**Conclusion:**

Arthrocentesis is useful in distinguishing streptobacillary septic arthritis from reactive arthritis of rat-bite fever. The sole use of commercial media containing sodium polyanethol sulfonate may render the bacterial culture negative. A detailed history of possible exposure to rodents should be elicited from patients with arthritis in order to facilitate microbiologic diagnosis.

## Background

Rat bites were known to result in human diseases for more than 2000 years. The causative agent of rat-bite fever named *Streptomyces muris ratti *was first isolated in 1914 by Schottuller [1]. It was a pleomorphic Gram-negative bacillus that colonizes the nasopharnyx of rats and other rodents [2]. Various names have been associated with this organism [3], and its nomenclature was finally changed to *Streptobacillus moniliformis*, which is the name used today, when the bacterium was isolated from a laboratory worker by Levaditi in 1925 [4]. The streptobacillary rat-bite fever is a systemic disease typically presenting with fever, skin rash, and arthralgia, with minimal inflammatory reaction over the bitten site [5]. Oral ingestion of the organism causes the disease Haverhill fever (also known as erythema arthriticum epidemicum), which is clinically similar to rat-bite fever and was associated with three reported large scale outbreaks [6-8]. Complications of the infection include endocarditis, pneumonia and metastatic abscesses [5]. With increased urbanization, the disease has become less common. However, a report from the Centers for Disease Control and Prevention in 2005 describing two rapidly fatal cases of rat-bite fever in previously healthy adults illustrates that rat-bite fever is still an important zoonotic infection after occupational and recreational exposure to rats, and *S. moniliformis*-related infections may be re-emerging [9].

While arthralgia is a common and typical presentation of rat-bite fever, we believe streptobacillary septic arthritis should be managed as a separate disease entity caused by *S. moniliformis *due to the differences in clinical manifestations, even though the clinical presentations of the two disease entities overlap and may not be easily distinguished from one another. There are several reviews on streptobacillary rat-bite fever in pediatric and adult patients and a review on *S. moniliformis *endocarditis [10-14], but none on streptobacillary septic arthritis. We hereby describe a patient with streptobacillary septic arthritis after rat bite, and review the literature on reported cases of streptobacillary septic arthritis, illustrating the differences between streptobacillary rat-bite fever and septic arthritis. The characteristics, treatment, and prognosis of *S. moniliformis *septic arthritis are also discussed.

## Case presentation

### Case report

A 58-year-old housewife presented to our hospital with five days' history of fever and polyarthralgia involving bilateral wrists, elbows, ankles and the right knee. She had a past history of osteoarthritis of both knees for 10 years not requiring any regular treatment. On admission, she had a blood pressure of 128/68 mm Hg, a pulse rate of 94 beats per minute, and a temperature of 37.8 oC. She appeared comfortable and not in distress, but all the affected joints were tender, swollen, with limited range of movement, especially over the left wrist and right knee. No cardiac murmurs were detected. The initial laboratory results revealed a normal peripheral leukocyte count of 7 500 cells/μL (77% neutrophils), hemoglobin level of 11.9 g/dL and platelet count of 191 000/μL. The liver and renal functions were all normal, and the urate level was 180 μmol/L. C-reactive protein was elevated to 38.3 mg/dL. X-ray of the left wrist and right knee did not show any juxta-articular erosion or loss of cartilages.

**Figure 1:**
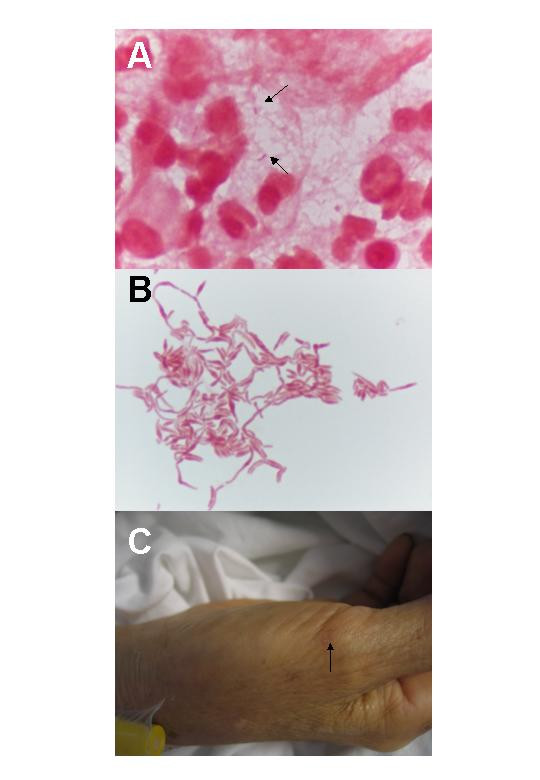
**Gram stain morphology of *Streptobacillus moniliformis *and patient's skin lesion**. (A) Gram stain appearance of synovial fluid showing Gram negative bacilli and numerous polymorphs (1000× magnification). (B) Gram stain appearance of *Streptobacillus moniliformis *after passaging showing filamentous Gram negative bacilli with bulbous swellings arranging into chains and clumps. (C) Rat bite mark (arrow) over base of right thumb 10 days after being bitten.

The patient was empirically treated as gouty arthritis with colchicine for four days without any clinical improvement, and the leukocyte count further increased to 13 700 cells/μL (91% neutrophils). Arthrocentesis of the right knee was performed with 20 mL turbid synovial fluid being aspirated. The total cell count of joint fluid was 14 850 cells/mL, predominantly polymorphs; no crystals were seen. Gram stain of the joint fluid showed regular Gram negative bacilli (Figure 1A) and became culture-positive after 48 hours of incubation at 37°C in 5% CO_2_. The organism only grew on 5% horse blood agar but not on chocolate or MacConkey agars, with characteristic pleomorphic filamentous Gram stain morphology and central bulbous swellings arranging into coils upon subculture (Figure 1B). It was identified as *Streptobacillus moniliformis *with compatible biochemical reactions (negative in the oxidase and catalase tests, unable to reduce nitrate to nitrite, and failed to produce indole from tryptophan). Upon further questioning, the patient recalled being bitten by a brown rat five days before the onset of joint pain when she traveled to a village in southern China. A healed wound with hyperpigmentation was noted over the right thumb base, which indicated the site of rat bite (Figure 1C). The patient was treated with intravenous ampicillin 1.5 g every six hourly. Her blood culture taken before starting antibiotics remained sterile after seven days of incubation. Due to persistent swelling and tenderness, an open arthrotomy of the left wrist was performed two days after starting antibiotic, which showed acute pyogenic synovitis. The patient was given 21 days of antibiotic. Upon discharge, she was afebrile, and the arthritis resolved completely without any long-term sequalae.

### Review of published cases

We searched PubMed (January 1970 to March 2006) for English-language articles that addressed streptobacillary septic arthritis. The keywords "Streptobacillus" and "septic arthritis" were used. Relevant additional cases were included from secondary references. Our reported case was also included in the review. Patient demographics, underlying diseases, types of rat exposure (if any), clinical presentation of the streptobacillary septic arthritis, site(s) of positive cultures, therapeutic interventions and outcome of treatment were recorded for all cases.

Twelve cases of *S. moniliformis *septic arthritis are reviewed including the present report and 11 previously published cases [15-25]. The clinical details of these cases are summarized in the table 1.

**Table 1:** Summary of reported cases of *Streptobacillus moniliformis *arthritis.

Study	Age in years, sex	Underlying diseases	Rat exposure	Potential contact with rats	Clinical presentation other than joint pain	Joint(s) involved	Treatment	Duration in days	Outcome
Mandel [15]	77, M	NR	Bite	Occupation (farmer)	Fever, anorexia, skin pustule	Both ankles and wrists, left sternoclavicular joint	Cloxacillin and ampicillin; penicillin G	2 14	Cured
Anderson & Marrie [16]	79, M	Oosteomyelitis of hands, Paget's disease	Bite	Occupation (farmer)	Fever and chills	Both knees	Penicillin G; penicillin V	28 14	Cured
Rumley et al. [17]	48, M	NR	None	Occupation (warehouse fork lift operator)	Fever, headache, cough, hoarseness, sore throat	Left wrist and right shoulder	Nafcillin and gentamicin	28	Cured
Holroyd et al. [18]	59, M	Alcoholism, pulmonary tuberculosis, chronic seizure disorder	None	NR	None	Small joints of hand, wrist, both knees, ankles and shoulders	Ticarcillin and gentamicin; penicillin G	NR 10	Cured
Fordham et al. [19]	63, M	NR	None	Occupation (pig farmer)	Fever, rash, anorexia, malaise	Both ankles, right knee	Flucloxacillin; vancomycin	NR	Cured
Hockam et al. [20]	12, M	None	Scratch	Pet	Fever, rash, skin pustule	Right hip	Arthrotomy, cefuroxime; ceftriaxone	21	Cured
Downing et al. [21]	13, M	None	Bite	Pet	Fever	Right hip	Arthrotomy; penicillin G; amoxicillin	42	Cured
Stehle et al. [22]	72, M	Osteomyelitis of knees with total knee replacement bilaterally	Bite	Animal lover, picking up rat near trashcans	None	Right elbow, 3rd left metacarpophalangeal, both knees	Antibiotics	NR	Cured
Wallet et al. [23]	56, F	NR	Bite	Pet	None	Right knee, left ankle, left shoulder, left elbow, left wrist	Penicillin G and ofloxacin; clindamycin and rifampin	NR	Cured
Thong & Barkham [24]	62, M	NR	Bite	NR	None	Both wrists, small joints of hands, right knee, right ankle, left foot	Ciprofloxacin and doxycycline; penicillin G	NR 28	Cured
Legout et al. [25]	60, F	Carcinoma of breast	Bite	Occupation (pet shop employee)	None	Both knees, elbows, ankles, hands	Penicillin; rifampin and clindamycin	28	Cured
Current case	58, F	Osteomyelitis of knees	Bite	Traveling	Fever	Left wrist and right knee	Arthrotomy; cloxacillin; ampicillin	3 21	Cured

NR, not reported.

The mean patient age was 72.0 years (median 72; range 12 to 79 years), and only two cases (16.7%) were under the age of 16 years; the male-to-female ratio was 3:1. Five patients (41.7%) had underlying diseases, with three of them reported to have osteoarthritis. Other underlying illnesses included carcinoma of the breast and chronic alcoholism. Eight patients (66.7%) developed streptobacillary septic arthritis after being bitten by rats, one patient acquired the disease after a rat scratch, and three patients could not recall any exposure or contact with rats or rodents. However, these three cases might still have potential contact with rats as two of them worked in rural areas (one as pig farmer and one as warehouse fork lift operator). The other patient was a chronic alcoholic, which could have exposed to rat bites and scratches when he was drunk. The most common cause for possible rat exposure was occupation (5 cases, 41.7%), followed by keeping of pet rats (3 cases, 25.0%), One patient sustained rat bite when the patient tried to pick up a rat near trashcans bare-handedly. Our patient was bitten by rat when she traveled to village in China.

Joint pain is the most common clinical presentation of streptobacillary septic arthritis in our series (100%). It is followed by fever in seven cases (58.3%). Two patients also reported to have skin rash, and another two patients had skin pustules (16.7%). Ten out of the twelve patients (83.3%) had polyarticular involvement. The most common joints associated with streptobacillary septic arthritis were the knees in seven patients (58.3%), ankles in six (50%), wrists in five (41.7%), small joints of hands in four (33.3%), elbows in three (25.0%), shoulders in three (25.0%), hips in two (16.7%), and sternoclavicular joint in one (8.3%). All patients had the bacterium isolated from joint fluid aspirate or synovial tissue, but only one of the seven patients with blood cultures taken had simultaneous *S. moniliformis *bacteremia. Three patients received arthrotomy together with antibiotic treatment. Details of the antibiotic regime was lacking in one. Seven patients received intravenous penicillin G (58.3%). For the remaining four patients, one received ampicillin (our patient), one was treated with nafcillin plus gentamicin, one was given ceftriaxone, and one was treated with flucloxacillin and then vancomycin. The outcome of the disease was good: all patients were cured without any mortality or long-term complication.

## Discussion

*S. moniliformis *is a facultatively anaerobic pleomorphic Gram-negative bacterium which appears as coccobacilli when first isolated from clinical specimens, but elongates to form intertwining wavy filaments with central swelling upon subculture. The optimal condition for isolation of *S. moniliformis *is culturing blood and body fluid on blood or tryptose soy agar enriched with 10–20% rabbit serum and incubated in a microaerophilic atmosphere with additional CO_2 _and humidification [26]. It may develop into cell wall-deficient L-form with typical "fried-egg" colonies in older cultures, which are more easily seen when subculture onto a clear medium [27]. When grown in broths (brain-heart infusion broth with cysteine 0.05% and Panmede 2.5%), whitish "puff-balls" and flocculated sediment can be seen at the bottom [28]. Growth of *S. moniliformis *can be inhibited by sodium polyanethol sulfonate present in many commercial blood culture media. Reports showed that the organism can still be isolated in culture medium containing up to 0.02% sodium polyanethol sulfonate [13,34], but a concentration of 0.035–0.05% is often found in systems such as BACTEC (BD, Sparks, MD) and BacT/Alert (Biomérieux, Durham, NC). Therefore, synovial fluid specimens from patients with septic arthritis, especially in those with a history of rat exposure, should not be submitted for culture solely in commercial blood culture media. New methods have been developed to help identifying *S. moniliformis*. These methods include gas-liquid chromatography analyzing the cellular fatty acid profile of the isolate [18,29,30], and molecular methods like 16S rRNA sequencing and polymerase chain reaction (PCR) amplification [23,31,32]. These molecular methods can also be used in typing isolates of *S. moniliformis *from humans and rodents, and thus identifying the source of infection even in the absence of known rat bite [33].

The classical presentation of streptobacillary rat-bite fever and Haverhill fever is fever, rash, arthralgia, myalgia, and *S. moniliformis *bacteremia [35]. It is believed that the joint symptoms may be reactive and autoimmune-mediated, which may persist for months to years. On the other hand, as shown in our series of infective arthritis due to *S. moniliformis*, fever and systemic upset was only present in half of the patients; rash was rare, and concomitant bacteremia was noted in one patient only. The clinical differentiation of reactive from infective streptobacillary arthritis is not always straightforward. A positive bacterial culture is essential to confirm the infective nature of the disease. The underlying factors that lead to these different types of manifestations are not known. We suspect that underlying joint abnormalities, such as Paget's disease and osteoarthritis, increase the risk of developing localized streptobacillary septic arthritis. Further studies are needed to unveil the mechanisms of articular invasion by *S. moniliformis*.

As seen in other forms of septic arthritis, the synovial fluid of *S. moniliformis*-infected joints would have increased number of polymorphs, and Gram smear may reveal the presence of micro-organisms. However, instead of being a monoarthritis, streptobacillary septic arthritis more commonly involves multiple joints and is usually asymmetrical. Peripheral joints are more likely to be affected than the axial joints with the knee joint being the most common site of involvement. This finding is compatible with another recently published review by Dendle *et al. *[36]. However, small joints of hands, which are rarely involved in septic arthritis other than those resulted from penetrative animal bites, may also be involved. In children, the hip joint is more commonly infected than the knee joint. The differential diagnoses of streptobacillary septic arthritis include *Spirillum minus *infection, which is another organism associated with rat bite fever, crystalline joint disease, gonococcemia, leptospirosis, brucellosis, viral arthritides, Hantavirus infection, Rocky Mountain spotted fever and other rickettsial infections. The differentiation of streptobacillary septic arthritis from reactive arthritis associated with rat-bite fever or other diseases is important as this may affect the choice and duration of antibiotic treatment. Last but not the least, autoimmune-mediated and reactive arthritides such as rheumatoid arthritis and Reiter's syndrome are also important differential diagnoses to be considered in patients presented with polyarthritis. If local and systemic corticosteroids are wrongly prescribed in patients with septic arthritis, this may lead to fatal sepsis. A joint fluid aspirate sent for cell count, microscopy, culture and other microbiological testing would be most useful in differentiating the above-named conditions and provide guidance for appropriate treatment.

Children and teenagers are reported to be more susceptible to have rat-bite fever due to their increased contacts with pet rodents (mice, guinea pigs and gerbils) [10,11,14,35]. However, there are only two cases younger than 16 years in our series, presumably because underlying chronic joint abnormalities are less common in this age group. It is believed that there is evidence of re-emergence of rat-bite fever or other *S. moniliformis*-associated diseases due to increased keeping of rats and rodents as pets not only by children but also by young adults, as illustrated in the fatal rat-bite fever case in Washington [9]. In addition, occupational exposure poses risk in adult patients. As revealed in our series, working in farms, barns, warehouses and pet shops are all associated with possible rat exposure even if the victims cannot recall any rat bite or scratch. Occupational exposure to rats also explains the relative predominance of the infection in male patients. Social behavior such as chronic alcoholism may be associated with rat exposure when one gets drunk. On the other hand, our patient illustrates the possibility of rat exposure during recreational activities such as travelling to villages and the countryside. Hence, occupational, social and recreational history should be sought in suspected cases of streptobacillary infections for possible rat contacts. Besides *S. moniliformis*, other infections can also be associated with rat bites and contacts. These include Hantavirus infections, leptospirosis, yersiniosis, *Spirillum minus *infections, and wound infections. Thus, individuals should seek medical attention immediately and report the exposure history after rat bites.

*S. moniliformis *is susceptible to multiple antibiotics, including penicillins, cephalosporins, carbapenems, erythromycin, clindamycin, tetracycline, teicoplanin and vancomycin, and intermediately susceptible to aminoglycosides and fluoroquinolones [35,37]. The recommended treatment for streptobacillary rat bite fever is intravenous penicillin G 1.2 million units per day for five to seven days followed by oral penicillin or ampicillin 500 mg four times a day for seven days. Alterative regimens for patients with penicillin allergy and infection due to L-form *S. moniliformis *include oral tetracycline 500 mg four times a day or intramuscular streptomycin 7.5 mg/kg twice daily [4,38]. Nonetheless, in case of streptobacillary septic arthritis, the choice of antibiotics is also governed by the ability of the drugs to penetrate the inflamed synovium and to achieve a desirable minimum inhibitory concentration in the synovial fluid. Aminoglycosides may not be useful in treating streptobacillary infective arthritis without bacteraemia because their diffusion into synoival fluid is suboptimal. As shown in the reported cases, patients also responded to nafcillin, flucloxacillin, vancomycin, clindamycin, and rifampin, and all of them have good recovery. Further studies are needed to determine the best drug for treating *S. moniliformis*-associated septic arthritis. Other treatment modalities such as arthroscopy, arthrotomy and joint lavage are important in controlling localized disease inside the joint, and are recommended for all patients with septic arthritis of large joints [39]. Arthroscopy is particularly essential for pediatric patients as it is less invasive than arthrotomy, and yet allows direct visualization of the joint structure and evaluates the degree of destruction, which may affect future growth in children [39-41]. The polyarticular presentation of streptobacillary septic arthritis may delay arthrotomy, but if the clinical response to antibiotics alone is suboptimal, surgical intervention is important for local drainage to remove any inoculations and reduce the bacterial load in the joint.

As compared to streptobacillary endocarditis which has a mortality rate of 53% [14], the prognosis of streptobacillary septic arthritis is good. All patients were cured without long-term complications. In patients with streptobacillary endocarditis, they may present with polyarthralgia and increased polymorphs in the joint fluid; but *S. moniliformis *would be isolated from blood rather than the joint fluid [14]. On the other hand, in our series of streptobacillary septic arthritis with good prognosis, *S. moniliformis *is isolated from the synovial fluid rather than the blood.

## Conclusion

Streptobacillary septic arthritis is an infection caused by *S. moniliformis *differing from traditional rat-bite fever and Haverhill fever after exposure to rats or other rodents. Asymmetrical polyarticular involvement of the peripheral joints is typical for streptobacillary arthritis. In contrary to streptobacillary rat-bite fever, skin rash and bacteremia is uncommon. Joint aspiration is important in making the diagnosis. Repeated examination of the bacterial culture plus recognition of the characteristic microscopic morphology is essential for identification of this bacterium. Early diagnosis requires a high index of suspicion and eliciting the relevant occupational, recreational, animal and pet exposure histories. Penicillin is the current recommended treatment, but the best regime is yet to be determined. Surgical intervention is essential in pediatric patients and those failed to respond to antibiotic treatment alone, and is associated with good outcome.

## Competing interests

The author(s) declare that they have no competing interests.

## Authors' contributions

TKFW carried out the literature review and drafted the manuscript. SSYW conceived of the literature review and helped to draft the manuscript. Both authors read and approved the final manuscript.

## Pre-publication history

The pre-publication history for this paper can be accessed here:


